# A Distinct Cytokine Profile and Stromal Vascular Fraction Metabolic Status without Significant Changes in the Lipid Composition Characterizes Lipedema

**DOI:** 10.3390/ijms22073313

**Published:** 2021-03-24

**Authors:** Stefan Wolf, Jeremy W. Deuel, Maija Hollmén, Gunther Felmerer, Bong-Sung Kim, Mauro Vasella, Lisanne Grünherz, Pietro Giovanoli, Nicole Lindenblatt, Epameinondas Gousopoulos

**Affiliations:** 1 Department of Plastic Surgery and Hand Surgery, University Hospital Zurich, 8091 Zurich, Switzerland; stefan.wolf2@usz.ch (S.W.); bong-sung.kim@usz.ch (B.-S.K.); Mauro.Vasella@usz.ch (M.V.); Lisanne.Gruenherz@usz.ch (L.G.); Pietro.Giovanoli@usz.ch (P.G.); Nicole.Lindenblatt@usz.ch (N.L.); 2 Division of Internal Medicine, University Hospital of Zurich, 8091 Zurich, Switzerland; jd862@cam.ac.uk; 3 MediCity Research Laboratory, University of Turku, 20520 Turku, Finland; maijal@utu.fi; 4 Division of Plastic Surgery, Department of Trauma Surgery, Orthopaedics and Plastic Surgery, University Medical Center Göttingen, Georg-August-University, 37099 Göttingen, Germany; gunther.felmerer@med.uni-goettingen.de

**Keywords:** lipedema, adipose tissue, lipidomics, mitochondrial respiration

## Abstract

Lipedema is an adipose tissue disorder characterized by the disproportionate increase of subcutaneous fat tissue in the lower and/or upper extremities. The underlying pathomechanism remains unclear and no molecular biomarkers to distinguish the disease exist, leading to a large number of undiagnosed and misdiagnosed patients. To unravel the distinct molecular characteristic of lipedema we performed lipidomic analysis of the adipose tissue and serum of lipedema versus anatomically- and body mass index (BMI)-matched control patients. Both tissue groups showed no significant changes regarding lipid composition. As hyperplastic adipose tissue represents low-grade inflammation, the potential systemic effects on circulating cytokines were evaluated in lipedema and control patients using the Multiplex immunoassay system. Interestingly, increased systemic levels of interleukin 11 (*p* = 0.03), interleukin 28A (*p* = 0.04) and interleukin 29 (*p* = 0.04) were observed. As cytokines can influence metabolic activity, the metabolic phenotype of the stromal vascular fraction was examined, revealing significantly increased mitochondrial respiration in lipedema. In conclusion, despite sharing a comparable lipid profile with healthy adipose tissue, lipedema is characterized by a distinct systemic cytokine profile and metabolic activity of the stromal vascular fraction.

## 1. Introduction

Lipedema is an adipose tissue disorder characterized by the disproportionate increase of subcutaneous fat tissue. The disease almost exclusively affects women in family clusters and onset is frequently associated with phases of hormonal changes, such as puberty, pregnancy and menopause. The disproportional fibroadipose tissue accumulation around legs and sometimes arms can lead to considerable disability, daily functioning impairment and psychosocial distress [1]. Due to the lack of epidemiological studies the exact prevalence is unknown. Nonetheless, rates are estimated at a considerable 7–15% [2,3]. Neither histopathological nor molecular hallmarks exist; thus, a large number of patients remain undiagnosed or misdiagnosed as other clinical entities, such as obesity and lymphedema.

The accumulation of adipose tissue characterizing lipedema is a result of the proliferation of adipose stem cells and hypertrophy of adipocytes, leading to a distinct lipedema phenotype [4]. Recent research suggests that lipedema adipose derived stems cells (ASCs) have a higher adipogenic differentiation potential compared to control ASCs which might contribute to the disease pathogenesis [5]. Previous work has demonstrated slight differences in the systemic lipid metabolism in lipedema with systemic lipid values ranging between the upper physiological range and slight pathological range [6]. The biological interpretation of these findings necessitates further research to unravel potential differences and thus potential biomarkers in the lipid composition between lipedema and control patients, both locally and systemically.

Recent studies conducted by multiple research groups, including ours, have revealed a distinct immune cell composition in lipedema characterized by increased macrophage infiltration whereas the T-cell compartment remains unchanged [7,8]. Interestingly, an M2 macrophage polarization phenotype was identified, attributed to CD163 overexpression [8]. The presence of such an immune signature is commonly associated with changes in cytokine levels. Initial investigations showed increased systemic levels of vascular endothelial growth factor (VEGF)-C which might be related to the increased macrophage infiltrate. In addition, downregulation of lymphatic-related cytokines such as Tie2, VEGF-A and VEGF-D which could be linked to a modified vascular permeability, developed secondarily to lipedema progression [8].

As the underlying pathophysiological mechanisms remain unclear, the identification of biomarkers would facilitate the timely diagnosis and treatment of the affected patients at an early stage. Therefore, this work investigates the fat tissue and serum in women suffering of lipedema, focusing on the alterations in local and systemic lipid composition, systemic cytokine profile changes and the stromal vascular fraction’s metabolic phenotype to gain inside into the pathophysiology of lipedema and potentially identify reliable biomarkers.

## 2. Results

### 2.1. Adipocyte Hypertrophy Characterizes Lipedema

**Figure 1:**
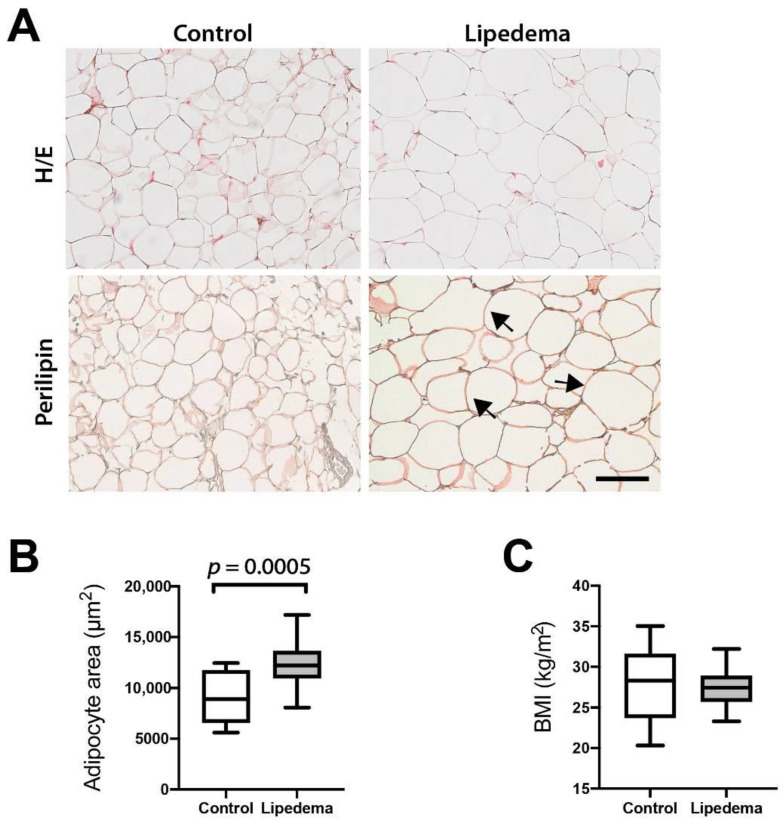
Adipocyte hypertrophy in lipedema. (**A**) Hematoxylin/eosin (H/E) and Perilipin immunostaining of paraffin-embedded adipose tissue demonstrating that even the largest adipocytes remain perilipin positive. The arrows indicate the large perilipin-positive adipocytes. Scale bar: 100 μm. (**B**) Quantification of the adipocyte size using the hematoxylin/eosin sections reveals adipose tissue hypertrophy. (**C**) The body mass index (BMI) values of the control and lipedema patients appear comparable. N (Control): 10 patients and N (Lipedema): 20 patients. The *p*-values indicate the statistical significance in comparison to the control (two-tailed Student t-test).

Previous work of our laboratory and others has revealed increased adipocyte size in lipedema (L) versus control (C) samples in anatomically matched tissue biopsies. To verify this finding, we analyzed the adipocyte size measuring the adipocyte cell area (in μm^2^) in hematoxylin/eosin stained sections of adipose tissue samples in a larger group of lipedema patients (C *n* = 10, L *n* = 20) using control patients with comparable BMI. We confirmed that lipedema patients exhibited significant adipose tissue hypertrophy compared to anatomically and BMI matched (C: 27.9 ± 4.2 kg/m^2^, L: 27.55 ± 2.5 kg/m^2^) patients as seen in Figure 1A–C (C: 8874 ± 2386 μm^2^ vs. L: 12,349 ± 2113 μm^2^). We used perilipin staining to verify that the structures quantified were indeed adipose cells and not lipid droplets (Figure 1A).

### 2.2. Lipid Mass Spectrometry Analysis Reveals a Comparable Lipid Composition in the Tissue and Serum of Lipedema Versus Control Patients

As adipocyte tissue hypertrophy appears to be a characteristic of lipedema we then evaluated whether this might reflect into the lipid composition in lipedema tissue or serum. The oily phase of the lipoaspirate as well as serum of patients (after 8 h of starvation) was analyzed by mass spectrometry.

**Figure 2:**
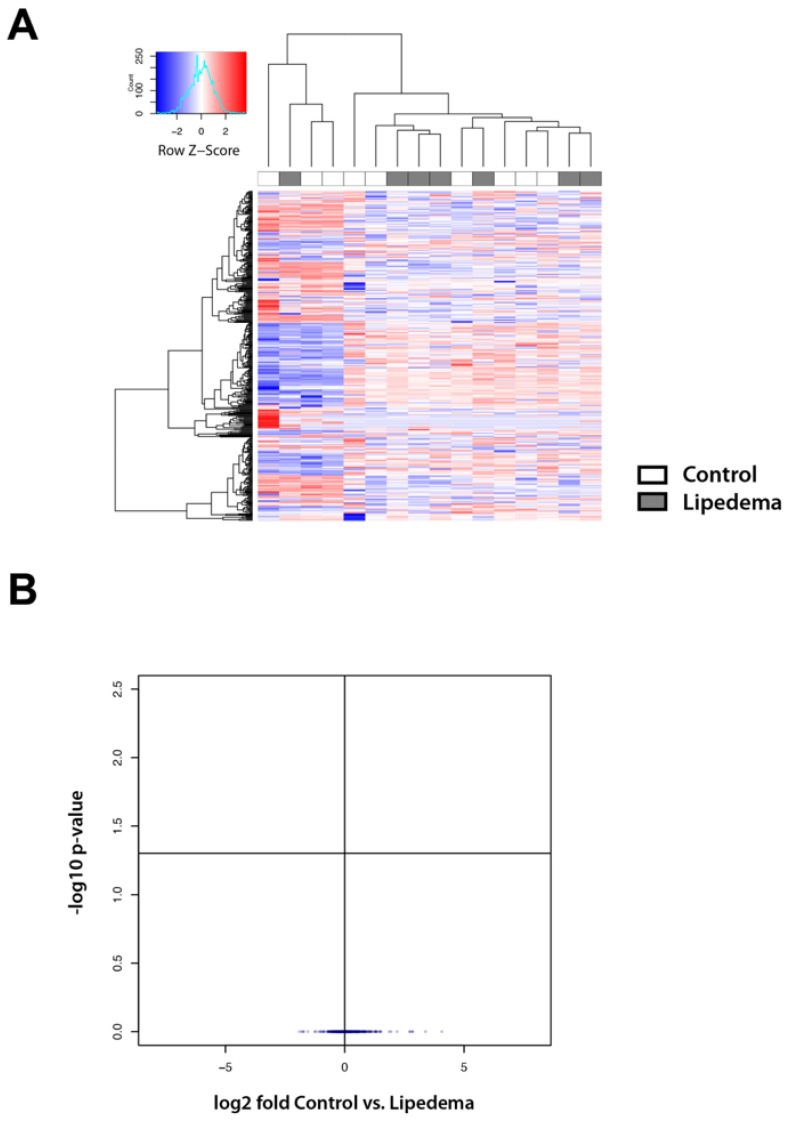
Lipid mass spectrometry analysis reveals a comparable lipid composition in the tissue of lipedema versus control patients (**A**) Lipidomic heat map showing log2 fold change of molecular lipid species comparing tissue of lipedema versus control patients using unsupervised hierarchical clustering. Each horizontal row represents a molecular lipid and each vertical column represents a tissue sample. Relative change of each lipid is indicated by coloring, and the scale is represented in the color key. (**B**) Volcano plot showing no statistically significant alterations in the lipid composition between the disease and the control groups. N (Control): 7 patients and N (Lipedema): 9 patients.

**Figure 3:**
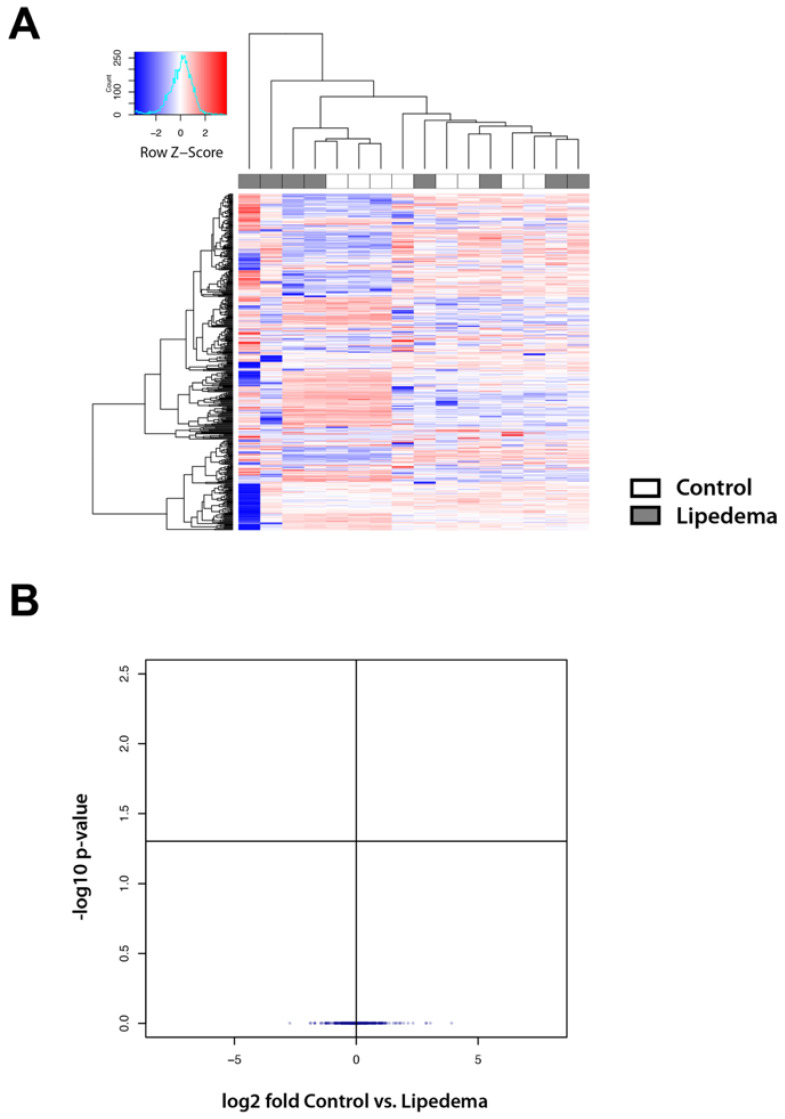
Lipid mass spectrometry analysis reveals a comparable lipid composition in the serum of lipedema versus control patients (**A**) Lipidomic heat map showing log2 fold change of molecular lipid species comparing serum of lipedema versus control patients using unsupervised hierarchical clustering. Each horizontal row represents a molecular lipid and each vertical column represents a serum sample. Relative change of each lipid is indicated by coloring, and the scale is represented in the color key. (**B**) Volcano plot showing no statistically significant alterations in the lipid composition between the disease and the control groups. N (Control): 8 patients and N (Lipedema): 8 patients.

The lipoaspirate and serum of 10 control and 10 lipedema patients was included in the study. Finally, lipoaspirate from 7 control and 9 lipedema patients and serum of 8 control and 8 lipedema patients were included into the analysis, excluding samples that showed visual contamination with blood (leading to lipid oxidation) [9]. The analysis of the oily phase of the aspirate and the serum revealed no statistically significant alterations in the lipid composition between the disease and the control groups (Figure 2A,B and Figure 3A,B).

### 2.3. A Distinct Cytokine Profile Characterizes Lipedema

**Figure 4:**
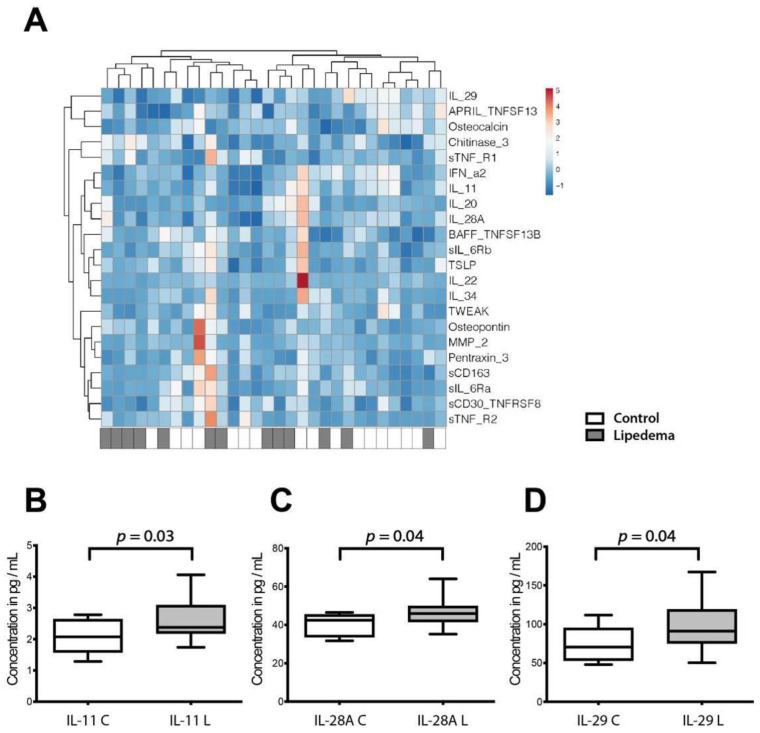
Inflammatory cytokine and chemokine profile of lipedema and control patients. (**A**) Multiplex immunoassay using serum samples from lipedema and control patients. Heat map showing log 2-fold changes in concentration of 22 cytokines/chemokines normalized by mean value of control samples. (**B**–**D**) Concentration of IL1 (**B**), IL28A (**C**), and IL29 (**D**) were significantly increased in the serum of lipedema patients. N (Control): 10 patients and N (Lipedema): 20 patients. The *p*-values indicate the statistical significance in comparison to the control (two-tailed Student t-test).

To dissect the differences of lipedema as compared to BMI-matched controls a broad analysis of the serum cytokine profile of lipedema versus control patients was performed using Multiplex. Thirty-seven cytokines were evaluated and 22 of them were detectable in the isolated serum (Figure 4A). The cytokines not detected in the serum included the following: interferons beta and gamma, (IFN-β, IFN-γ) interleukins 2, 8, 10, 12(p40), 12(p70), 19, 26, 27(p28), 32, 35 (IL-2, IL-8, IL-10, IL-12(p40), IL-12(p70), IL-19, IL-26, IL-27(p28), IL-32, IL-35), tumor necrosis factor superfamily 14 (TNFSF14) and the matrix metalloproteinases 1 and 3 (MMP-1, MMP-3). Out of the ones detected, IL-11 (C: 2.07 ± 0.51 vs. L: 2.64 ± 0.65 pg/mL; 95%CI: C: 1.65–2.68 vs. L: 2.31–2.96 pg/mL), IL-28A (C: 39.98 ± 5.61 vs. L: 46.17 ± 6.65 pg/mL; 95%CI: C: 35.41–44.57 vs. L: 42.98–49.37 pg/mL) and IL-29 (C: 74.13 ± 20.58 vs. L: 96.19 ± 29.1 pg/mL; 95%CI: C: 58.62–89.66 vs. L: 82.22–110.2 pg/mL) were significantly increased in the serum of lipedema patients (Figure 4B–D).

**Figure 5:**
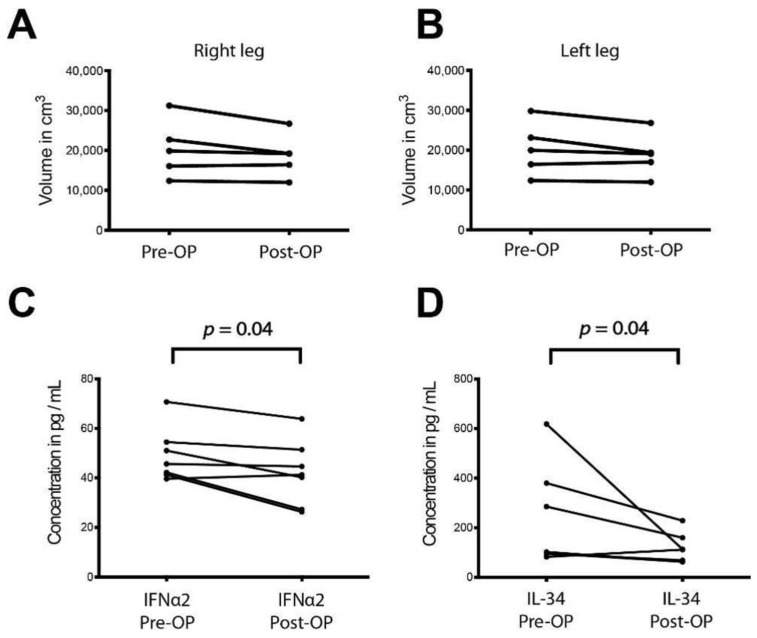
Inflammatory cytokine and chemokine profile in the serum of lipedema patients pre- and one year post-operatively (**A**,**B**) Analysis of the limb volume pre- and one year post-operatively observed no significant volumetric changes. N: 5 patients (**C**,**D**) Serum was examined pre-operatively and one year post-operatively using Multiplex. Out of 22 cytokines detected, interferon alpha (IFNα) and interleukin 34 (IL34) blood concentrations were decreased one year post-operatively. N: 7 patients. The *p*-values indicate the statistical significance in comparison to the control (two-tailed paired Student t-test).

As liposuction is currently the only treatment option for lipedema we then sought to investigate any potential systemic effects in the circulating cytokines upon surgical treatment. An analysis of the limb volume pre- and one year post-operatively was conducted first to demonstrate that any possible observed changes are not due to significant volume deviations at the two evaluated timepoints. Indeed, no significant volumetric changes were detected in the affected extremities pre-and post-operatively as depicted in Figure 5A,B. The serum of seven patients was examined directly pre-operatively and one year post-operatively using Multiplex. Out of the twenty-two detected cytokines, interferon alpha (IFNα) (Pre-OP: 49.29 ± 10.06 vs. Post-OP: 42.15 ± 12.17 pg/mL; 95%CI: Pre-OP: 39.24–59.35 vs. Post-OP: 29.99–54.32 pg/mL) and interleukin 34 (Pre-OP: 237.21 ± 188.97 vs. Post-OP: 115.6 ± 56.78 pg/mL; 95%CI: Pre-OP: 48.44–426 vs. Post-OP: 58.88–172.3 pg/mL) were found to be decreased one year post-operatively.

### 2.4. Increased Oxidative Metabolism Capacity of the SVF from Lipedema Patients

**Figure 6:**
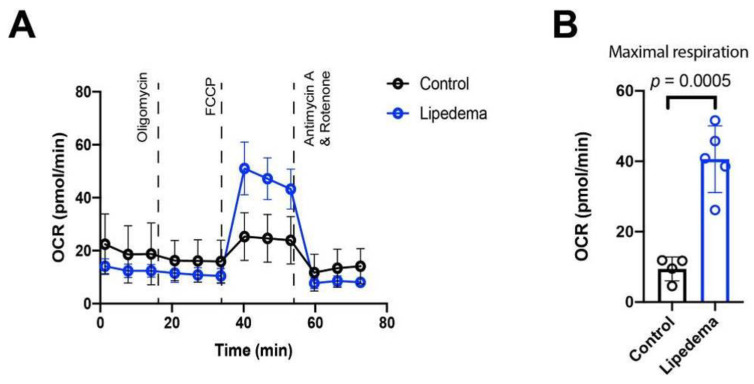
Oxygen consumption rates of stromal vascular fraction (0.15 × 10^6^ cells/well) of lipedema and controls patients. (**A**) At the basal respiration, the ATP (adenosine triphosphate) linked respiration, the non-mitochondrial respiration, no significant alterations were observed. Each data point represents mean value of 4 or 5 patients. (**B**) Maximal respiration revealed a 4-fold increase in lipedema patient compared to controls. Maximal respiratory capacity is derived by subtracting the ORC (oxygen consumption rate) value at time point 6 from the value at time point 7. Each data point represents mean value of one patient. N (Control): 4 patients and N (Lipedema): 5 patients. The *p*-values indicate the statistical significance in comparison to the control (two-tailed Student t-test).

After discovering a distinct cytokine profile in lipedema and knowing that inflammatory cytokines potentially influence metabolic activity of tissues, we set out to determine the metabolic status of the stromal vascular fraction (SVF) in lipedema and control patients. To evaluate the mitochondrial function/activity of the SVF we performed a mitochondrial stress test using an Agilent Seahorse XFe96 Analyzer. At the basal respiration (prior to addition of mitochondrial inhibitors/uncouplers) the ATP linked respiration (calculated after oligomycin administration) and the non-mitochondrial respiration (after antimycin A and rotenone administration) no significant alterations were observed. With the administration of the uncoupler carbonyl cyanide-4-(trifluoromethoxy)phenylhydrazone (FCCP) the maximal possible oxygen consumption was determined. The SVF of lipedema patients showed a highly increased and significant maximal respiration (OCR: C: 9.43 ± 2.95 pmol/min; L: 40.6 ± 8.48 pmol/min; 4-fold increase; and *p* = 0.0005) compared to controls, further suggesting that lipedema is associated with an enhanced mitochondrial function of the SVF (Figure 6).

## 3. Discussion

In the present study we examined the lipid composition in adipose tissue and serum of lipedema patients as well as the serum inflammatory cytokine profile and adipose tissue metabolic profile compared to gender-, anatomically- and BMI-matched control samples undergoing elective plastic surgery procedures.

A hallmark of lipedema is the profound morphologic remodeling of adipose tissue, exhibiting a prominent adipocyte hypertrophy. The present study confirms these results which were first demonstrated in previously published histological studies [6,7]. Changes, such as increased adipocyte size, often reflect metabolic states within those cells [10]. Our previous research confirmed an aberrant lipid metabolism and a distinct adipogenic gene expression profile. Furthermore, serum triglycerides, LDL and cholesterol levels were found to be slightly but also significantly increased with values ranging from the upper physiological to pathologic levels [6]. However, little is known about the lipids that comprise lipedema adipose tissue or circulate systemically in the serum. Therefore, we performed a lipidomic analysis of lipoaspirates and serum from lipedema and control patients and identified 400 lipids present in a similar abundance in both groups. Accordingly, our data suggest that lipedema is not a condition of fundamentally altered or grossly defective lipid metabolism. The slight changes in the serum lipids from previous studies could not be confirmed, possibly due to our study being underpowered.

These results clearly allow a differentiation of lipedema from lymphedema and obesity. In the lipidomic profile of plasma from obese patients a significant reduction in lyso-phosphatidylcholine levels is visible [11] which does not occur in lipedema. The analysis of lipid molecules in the oil of lipoaspirates from primary lymphedema patients exhibits a signature of increased cyclopropane-type fatty acids and inflammatory mediators’ arachidonic acid and ceramides. Interestingly C20:5 and C22:6 omega-3-type lipids were increased in the adipose tissue correlating with the duration of lymphedema. Nevertheless, lymphedema has a normal lipid profile containing a signature of inflammation and omega-3-lipids [12].

As a second hallmark, lipedema is associated with a state of low-grade inflammation. Our previous research showed that a distinct immune cell infiltration occurs in lipedema, characterized by the infiltration of M2 polarized macrophages [8]. The presence of such an immune signature is associated with systemic changes reflected in circulating cytokines’ levels. Only limited data of the cytokine profile in lipedema is available. To elucidate potential differences, we performed a Multiplex Immunoassay System to evaluate 37 circulating cytokines in the serum of lipedema versus BMI-matched control patients as well as lipedema patients pre- and one year post-operatively.

The comparison between lipedema and control patients (pre-operatively) revealed significantly increased IL-11, interferon type III family IL 29 (interferon lambda 1) and IL28A (interferon lambda 2) levels. IL-28A and IL-29 are secreted primarily by dendritic cells and macrophages [13] and induce antiviral responses and primarily act at anatomical barriers, including epithelial surfaces [14]. The third upregulated cytokine, IL-11, regulates adipogenesis by binding IL-11Rα receptor [15,16]. The primary source for IL-11 is the stromal vascular fraction [17] which is increased in adipose tissue of lipedema patients [4]. The alterations in circulating IL11, IL28A and IL29 levels are possibly linked to the specific immunological niche present in lipedema. 

It was of particular interest to evaluate the potential systemic effect of liposuction, the currently most widely accepted treatment for lipedema, in regard to the changes in circulating cytokines. Serum was isolated from blood samples taken after eight hours of starvation either directly before surgery or one year after surgery. INFα2 and IL34 were found to be significantly reduced in the patients after surgery. IL-34 is expressed in human adipose tissues by adipocytes, SVF and its circulating concentration is significantly elevated in obese patients. IL-34 is associated with obesity-induced inflammation and the pathogenesis of related diseases such as insulin resistance [18]. On the other hand, IFN-α inhibits adipocyte differentiation and lipid droplet accumulation [19] and can induce apoptosis in adipose tissue cells [20]. Macrophage-derived IFN-α, together with IL-12 and IL-18, efficiently induces IFN-γ expression [21]. Liposuction has been found to have persistent and long-term beneficial effects without relapse over up to 12 years [22]. It is not clear whether the altered cytokine milieu post-surgery could contribute to this beneficial effect or if it is the result of the reduced adipose tissue with its remarkable secretory capacity. In contrast to cosmetic liposuction the main goal of surgical treatment of lipedema is not the volume reduction but rather the reduction of the painful fat tissue accumulation linked with sensitivity to pressure, feeling of tension, bruising and general impairment to quality of life. Thus, the marginal volumetric changes in this patient group (with the moderate BMI values) following liposuction are not surprising. The underlying mechanisms by which liposuction may downregulate INFα2 and IL34 serum levels is a subject of further investigation.

It is known that circulating cytokines influence many cellular metabolic activities. The metabolic activity of the adipose tissue is particularly determined by the interplay between adipocytes, preadipocytes, endothelial cells and immune cells which are all present in the SVF and appear altered in lipedema tissue in terms of cell content and cellular subtype composition [4]. To evaluate the metabolic activity of the adipose milieu, we analyzed SVF cells isolated from liposuction material of lipedema and control patients. The isolated cells from lipedema showed a higher oxygen consumption rate than control SVF under maximal respiratory stress (uncoupling), indicating that lipedema SVF has an increased oxidative metabolic capacity. These results point toward an increased activation state. An essential part of SVF consists of macrophages which have a potential role in lipedema. An altered macrophage metabolism not only is a characteristic of polarized macrophage subsets, it is also a prerequisite for proper polarization and inflammatory regulation. Notably, the inhibition of glycolysis or OXPHOS/FAO has been demonstrated to impair M1 or M2 activation [23,24]. Indeed, metabolic changes affect metabolite concentrations that are direct regulators of the macrophage phenotype [25,26]. 

The current study has been limited by the relatively low number of patients enrolled. The various test material was limited and sufficient amounts from every patient were not available to perform all experiments, resulting in differential number of patients (from the same cohort) being used for the various tests. These limitations were overcome by the very consistent and comparable characteristics of the selected and analyzed groups (anatomic location, age and BMI). Particularly, the sample size of the patients who were examined pre-and post-operatively was low and the alteration in the cytokine profile was slightly variable. These results ranged from massive downregulation to no changes in the post-surgery cytokine profile (Figure 5C,D) which could be attributed to individual differences. Nevertheless, a moderate but significant effect could be observed and these preliminary results need to be proven in a larger sample size.

Our results further increase the understanding of lipedema and underpin the distinct nature of the disease. Notwithstanding the characteristic adipose tissue hypertrophy, the lipid composition in lipedema is comparable to the controls. Serum lipid markers do not appear to be sufficient to define or diagnose the disease, suggesting that potential biomarker development should be based elsewhere. Interestingly, a distinct systemic cytokine profile is present in lipedema and our preliminary results offer an opportunity to understand better how the symptom relief after surgery mirrors certain systemic changes potentially linked to immunological components. So far there are no blood-based markers to diagnose lipedema. The alterations in the cytokine milieu, which need to be confirmed in larger patient cohorts, offer a promising opportunity for the development of lipedema biomarkers. The alterations in the SVF metabolism present an intriguing finding, suggesting a metabolically active adipose tissue. This requires further elucidation in regard to the mechanisms involved and particularly the role of the immune component in the regulation of the adipose tissue metabolism in lipedema. 

## 4. Materials and Methods

### 4.1. Patients

The protocols of the current study were approved prior to patient recruitment by the Ethical Committee of the University Hospital Goettingen, State of Lower Saxony, Germany (Nr. 23-11-17, accepted on 23. November 2017) and the study has been conducted according to the principles of the Declaration of Helsinki and its amendments. All patients were informed in detail prior to the surgical procedures in oral and written form and provided their written informed consent. The samples were obtained from lipedema and BMI- as well as age-matched control female patients. Lipedema was diagnosed based on the criteria of Wold et al. [27], namely (1) female, (2) bilateral increase of the adipose tissue of the lower extremities sparing the feet, (3) negative Stemmer’s sign, (4) pain, tenderness and a tendency to bruise in the affected extremities, (5) adiposity demonstrated resistance to attempts at weight loss or persistence to extremity elevation. All lipedema patients included met the aforementioned criteria. The tissue derived from the proximal part of the thigh, as anatomically matched biopsies. The patient characteristics of the study cohort are provided in the Table 1. The number of patients evaluated in each type of analysis is provided in the Appendix A Table A1.

### 4.2. Tissue Collection and Immunohistochemistry

During the operating procedure fat tissue specimens for histology were collected and fixed for 4 h in paraformaldehyde/phosphate-buffered saline (PBS) at 4 °C. Subsequently, the samples were embedded in paraffin.

For (immuno-)histological analysis and assessment of adipose tissue architecture the specimens were cut into 5-μm thick paraffin sections and stained at the Department of Pathology of the University Medical Center Goettingen according to standardized protocols. For the perilipin stain, paraffin-embedded sections were deparaffinized and rehydrated. Antigen retrieval was performed with proteinase K (Dako S3020), and endogenous peroxidase activity was blocked using Bloxall (Vector Laboratories, Burlingame, CA, USA; Vector SP-6000). After blocking (Vectastain Mouse-HRP-Kit; Vector PK-6102 plus 1.5% horse serum), the sections were incubated with guinea pig antihuman perilipin antibody (Fitzgerald Industries International, Acton, MA, USA; 20R-PP004, 1:200) at 4 °C overnight. After washing steps with PBS, bound antibody was visualized using the Vectastain Kit with DAB substrate, according to the manufacturer’s instructions.

Histology images were obtained using a Leica Leitz DM RXE microscope equipped with a Leica DFC490 camera, and up to five images per tissue were acquired using a PL Fluotar 20x/0.5 numerical aperture or PL Fluotar 40x/0.7 numerical aperture objective. Morphometric analysis of adipocyte characteristics was performed using ImageJ software (National Institutes of Health, Bethesda, MD, USA).

### 4.3. Serum Isolation

For the cytokine evaluation 10 mL of blood was collected in a S-Monovette (Sarstedt, Nuernbrecht, Germany) preoperatively and upon 8–10 (6–8 h) h of starvation and one year postoperatively. Blood was left to coagulate for 30 min at RT and then was centrifuged for 10 min at 1000× *g*. Serum was aliquoted and stored at −80 °C until usage.

### 4.4. Lipid Extraction

A total of 200 µL of the lipid containing sample was dispersed in 1.5 mL of methanol containing 50 µg/mL butyrated hydroxytoluene (BHT) in a glass tube with a PTFE (polytetrafluoroethylene) lid, both baked at 180 °C for 24 h prior to usage. A total of 5 mL of methyl tert-butyl ether (MTBE) was added and the tube was agitated at room temperature for one hour. Then, 1.25 mL of water was added and the tube was briefly mixed and then centrifuged for 10 min at 1000× *g*. The upper phase containing most of the lipids was then transferred to a new glass tube and stored at −80 °C for a max of 30 days [28]. All chemicals were obtained from Sigma (Sigma-Aldrich, St. Louis, MO, USA) at the highest available purity.

### 4.5. Mass Spectrometry

The solvent was removed under a stream of liquid nitrogen and the lipids were solubilized in 0.5 mL of methanol (MeOH). After shaking them for 20 min at room temperature, samples were diluted 5 times to a final concentration of 50% MeOH and directly injected into the liquid chromatography-mass spectrometry (UPLC-MS) system for further analysis. Lipids were separated on a nanoAquity UPLC (Waters) equipped with a HSS T3 capillary column (150 μm × 40 mm, 1.8 μm particle size, Waters). Buffer A was 5 mM ammonium acetate in 5% acetonitrile; buffer B was 5 mM ammonium acetate in 90% isopropanol and 10% acetonitrile. A 10-min gradient from 2% B to 98% B was applied at an initial flow rate of 3 μL/min, linearly decreased to 2.5 μL/min. The injection volume was 1 μL. The UPLC was coupled to a Q-Exactive MS (Thermo) by a nanoESI source. MS data was acquired using positive polarity and data-dependent acquisition (dd-MS2) in top-5 scan mode, over a mass range of 80 to 1200 m/z at a resolution of 70,000 for MS and 17,500 for MS2. Normalized collision energy was stepped from 20 to 30. All solvents used were of quality HPLC grade (Chromasolv, Sigma-Aldrich, St. Louis, MO, USA). Data was analyzed using Progenesis QI software (Waters Corportation, Milford, MA, USA) using the LipidMaps database (www.lipidmaps.org) and R statistical software (www.r-project.org). Complete lipidomic analysis is shown in the Supplementary Table S1.

### 4.6. Isolation of the Stromal Vascular Fraction

Adipose tissue was digested with 2 mg/mL collagenase dissolved in RPMI glutamax medium and was incubated at 37 °C under moderate shaking for 1 h. After centrifugation at 1000× *g* for 5 min, the cell pellet was incubated with erythrocyte lysis buffer for 10 min on ice to eliminate red blood cells. The cell suspension was diluted in PBS and the supernatant was aspirated after centrifugation for 5 min at 1000× *g*. The pellet was washed with PBS and filtered through a 70 μm cell strainer. After another centrifugation step at 500× *g* for 5 min, the supernatant was removed, and the isolated SVF cells were frozen in 5% FBS and 5% DMSO.

### 4.7. Multiplex Analysis

Cytokine quantification in the human serum samples was performed using Bio-Plex Pro Human Inflammation Panel 1, 37-Plex (Bio-Rad Laboratories, Hercules, CA, USA) according to the manufacturer’s instructions.

### 4.8. Measurement of SVF Mitochondrial Respiration

For the Mito Stress Test, SVF isolated from control and lipedema patient adipose tissue was plated at 0.15 × 10^6^ cells/well as four technical replicates (for each patient) in complete IMDM (L-glut, 10% FBS and penstrep) and left to adhere overnight on a Seahorse Assay Plate at 37 °C in a humified 5% CO2 incubator. IMDM was replaced with Seahorse Assay Medium pH 7.4 supplemented with 10 mM glucose, 2 mM L-glutamine and 1 mM sodium pyruvate and incubated for one hour at 37 °C in a non-CO_2_ incubator. Thereafter, the cells were transferred to the Seahorse XFe96 Extracellular Flux Analyzer (Agilent Technologies, Santa Clara, CA, USA) where the cells were treated sequentially with 1 µM oligomycin, 1 µM FCCP and 0.5 µM Rotenone/antimycin A and analyzed for mitochondrial respiration. Each technical replicate was normalized to the amount of DNA/well which was determined using the CyQuant kit (C35011, Thermo Fisher, Zug, Switzerland), according to the manufacturer’s instructions. A mean was calculated per patient from the technical replicates and then these values were used for the further statistical analysis. For the calculation of the maximal respiration, the ORC value at time point 6 was subtracted from the value at time point 7 and plotted separately.

### 4.9. Statistical Analysis

All data are expressed as mean ± SD; boxplot show the average and the 25–75 percentiles while the whisker plots exhibit the 5–95 percentiles. Outliers have been identified with the Grubb’s test and have been excluded from the analysis. A non-parametric unpaired Mann–Whitney U-test was performed for non-Gaussian distribution, whereas a two-tailed Student t-test was performed for Gaussian-distribution. The results of the statistical analysis in lipid spectrometry were corrected for multiple testing using the Benjamini-Hochberg method.

Sample sizes and statistical analyses are indicated in the figure legends, unless otherwise mentioned. Statistical analyses were performed using GraphPad Prism V8.0 (GraphPad Software, San Diego, CA, USA). *p* < 0.05 was accepted as statistically significant.

Table S1 shows the complete lipidomic analysis of the adipose tissue and serum of lipedema and control patients.

## Figures and Tables

**Table 1:** Patient characteristics.

Patient Characteristics	Study Cohort
Number of cases	30
Lipedema patients	20
Control patients	10
Gender	
Female	30
Male	0
Mean age (in years)	
Lipedema patients	48.65 ±.11
Control patients	49.8 ± 8.6
Mean BMI (in kg/m^2^)	
Lipedema patients	27.55 ± 2.45
Control patients	27.85 ± 4.2
Lipedema Staging	
Stage I	1
Stage II	10
Stage III	9
Stage IV	0

## Data Availability

Not applicable.

## Supplementary Materials

The following are available online at https://www.mdpi.com/1422-0067/22/7/3313/s1.

## Appendix A

**Table A1:** Number of patients used in each type of analysis.

Patient Characteristics	Study Cohort	Histological & Cytokine Analysis	Lipidomic Analysis	SVF Mitochondrial Respiration Analysis
Number of cases initially included	30	30	20	10
Lipedema patients	20	20	10 *	5
Control patients	10	10	10 *	5 **
Number of cases analyzed		30		
Lipedema patients		20	9 (aspirate) 8 (serum)	5
Control patients		10	7 (aspirate) 8 (serum)	4
Gender				
Female	30	30	20	10
Male	0	0	0	0

* Samples with visible amount of blood contamination were excluded. ** one control patient was excluded from the analysis as an outlier.
